# Palliative care or supportive care?

**DOI:** 10.1016/j.clinme.2025.100487

**Published:** 2025-07-14

**Authors:** Amy Taylor, Andrew Davies

**Affiliations:** a Trinity College Dublin, School of Medicine, Dublin, Ireland; b Academic Department of Palliative Medicine, Our Lady’s Hospice & Care Services, Harold’s Cross, Dublin, Ireland; c University College Dublin, School of Medicine, Health Sciences Centre, Dublin, Ireland

**Keywords:** Palliative medicine, Palliative care, Supportive care

## Abstract

Palliative care is the active holistic (physical, psychological, social and spiritual) care of people who experience health-related suffering due to severe illness and those close to them, aiming to improve their quality of life. There are three levels of palliative care: palliative care approach, generalist palliative care, and specialist palliative care. Traditionally associated with end-of-life care, this article highlights the evolution of specialist palliative care, now deemed to be applicable throughout the course of an illness. Terminologies surrounding ‘palliative care’ are also considered, especially the notable nomenclature debate between ‘palliative care’ and ‘supportive care’. Specialist palliative care has evolved somewhat over time, and will need to continue to evolve to maintain its relevance.

## Highlights

• Palliative care refers to the holistic care of people who experience health-related suffering and those close to them.
• Palliative care is relevant throughout the disease trajectory and not just at the end of life.
• Various terminologies are used interchangeably with ‘palliative care’, although these can have different meanings.
• Supportive care can be described as the prevention and management of the adverse effects of cancer and its treatment.
• Palliative care can be considered one part of supportive care.

## Introduction

The term ‘palliative’ has been around since the 15th century, and refers to something ‘that relieves the symptoms of a disease or condition without dealing with the underlying cause’.^1^ In contrast, the term ‘palliative care’ was only devised in the 1970s, and was initially employed to describe the application of hospice principles to hospital inpatients (and avoid the term ‘hospice care’).^2^ Subsequently, palliative care has been applied to relevant services operating in hospitals, hospices and community settings (eg home, care/nursing homes).

## Definition of palliative care

The definition of palliative care has evolved over time. The original World Health Organization definition specified the relevance of palliative care for individuals not responsive to curative treatment.^3^ This was updated in 2002 to ‘an approach that improves the quality of life of patients and their families facing the problem associated with life-threatening illness, through the prevention and relief of suffering by means of early identification and impeccable assessment and treatment of pain and other problems, physical, psychosocial or spiritual’.^4^ The International Association for Hospice and Palliative Care (IAHPC) noted ongoing differences in perceptions about ‘palliative care’ and developed a consensus-based definition that defines palliative care as ‘the active holistic care of individuals across all ages with serious health-related suffering due to severe illness and especially of those near the end of life. It aims to improve the quality of life of patients, their families and their caregivers’.^5^ A series of explanatory statements were also proposed to support the IAHPC definition (Box 1). Importantly, while the definition highlights individuals ‘near the end of life’, the explanatory statements include ‘is applicable throughout the course of an illness, according to the patient’s needs’. The latter reflects the change in scope of palliative care over time (Fig. 1).^6^

**1:**
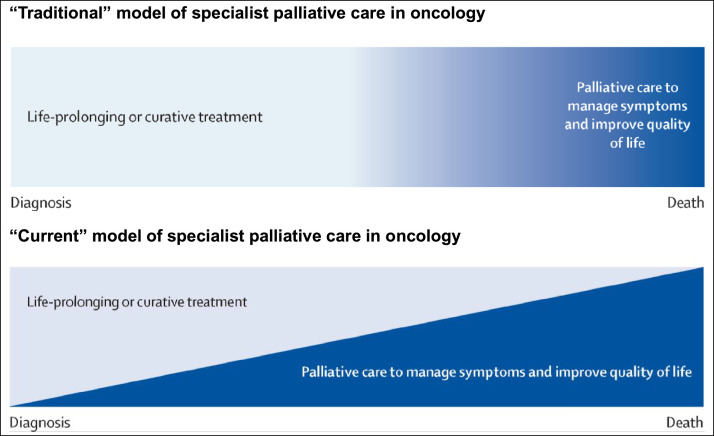
Traditional and current models of specialist palliative care in oncology (adapted from^6^).

Box 1 **Components of palliative care.5**

❖ ‘Includes, prevention, early identification, comprehensive assessment and management of physical issues, including pain and other distressing symptoms, psychological distress, spiritual distress and social needs. Whenever possible, these interventions must be evidence based.
❖ Provides support to help patients live as fully as possible until death by facilitating effective communication, helping them, and their families determine goals of care.
❖ Is applicable throughout the course of an illness, according to the patient’s needs.
❖ Is provided in conjunction with disease-modifying therapies whenever needed.
❖ May positively influence the course of illness.
❖ Intends neither to hasten nor to postpone death, affirms life, and recognises dying as a natural process.
❖ Provides support to the family and caregivers during the patient’s illness, and in their own bereavement.
❖ Is delivered recognising and respecting the cultural values and beliefs of the patient and family.
❖ Is applicable throughout all healthcare settings (place of residence and institutions) and in all levels (primary to tertiary).
❖ Can be provided by professionals with basic palliative care training.
❖ Requires SPC with a multiprofessional team for referral of complex cases.’^5^

Alt-text: Unlabelled box

## Palliative care ‘levels’

The aforementioned components of palliative care reflect ‘holistic care’, which is practised by many non-specialist healthcare professionals. Indeed, the European Association for Palliative Care (EAPC) has defined three levels of palliative care:^7^ 1) palliative care approach – provided by all healthcare professionals (integrating the general principles of palliative care/holistic care in their work); 2) generalist palliative care – provided by non-palliative care healthcare professionals with some relevant education; and 3) specialist palliative care – provided by dedicated palliative care healthcare professionals with relevant education, training and experience. Palliative medicine is the medical specialty related to specialist palliative care.^8^

## Evolution of specialist palliative care

Initially, the focus of palliative care was on patients with cancer, and those with a limited prognosis (ie end-of-life care). However, palliative care is increasingly involved in patients with life-limiting non-malignant diseases (eg motor neurone disease, chronic obstructive pulmonary disease, chronic cardiac failure, hepatic/renal failure). Moreover, palliative care has expanded its reach within oncology to include patients throughout the cancer journey, including patients receiving anticancer treatment (‘early’ palliative care),^9^ and even patients living beyond cancer (‘cancer survivorship’).^10^ Importantly, a Cochrane systematic review of early palliative care in patients with advanced cancer reported improved symptom control, and improved quality of life.^11^ However, palliative care is not necessarily associated with improved outcomes in all cohorts of patients (eg patients with dementia).^12^

## Supportive care

There are also different interpretations of the term ‘supportive care’. *The Lancet* Oncology Commission suggests that supportive care is relevant at all stages of the disease (including for those who have been cured), whereas palliative care is applicable only in life-limiting disease.^6^ Other organisations suggest that palliative care is one facet of supportive care. Indeed, the Multinational Association of Supportive Cancer in Cancer (MASCC) defines supportive care as ‘the prevention and management of the adverse effects of cancer and its treatment. This includes management of physical and psychological symptoms and side effects across the continuum of the cancer journey from diagnosis through treatment to post-treatment care. Supportive care aims to improve the quality of rehabilitation, secondary cancer prevention, survivorship, and end-of-life care’.^13^ Thus, supportive care includes palliative care, but is more than palliative care.^14^

**Fig. 2:**
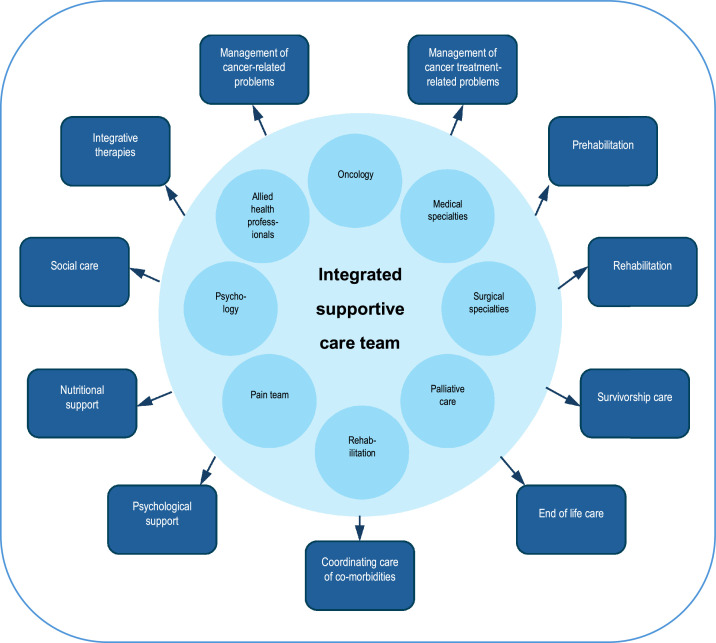
Multidisciplinary supportive care team.

As discussed, the term ‘palliative care’ was developed in part due to the negative (nihilistic) associations with the term ‘hospice care’. Many specialist palliative care teams have rebranded themselves ‘supportive care’ teams, or ‘supportive and palliative care’ teams, for similar reasons. Interestingly, studies suggest that such rebranding results in greater oncology referrals, and also earlier oncology referrals (to specialist palliative care teams).^15^ Some teams do indeed provide both palliative care and supportive care interventions under the same service, but in other cases the replacement of ‘palliative care’ with ‘supportive care’ potentially causes confusion, since ‘true’ supportive care provides a greater range of services and requires a wider multidisciplinary approach. Fig. 2 demonstrates the ideal integrated supportive care service, where a range of historically independent healthcare teams/services (including specialist palliative care) coordinate care in a unified model of collaborative teamwork.^16^ Indeed, most specialist palliative care professionals have limited training/experience in areas such as managing anticancer treatment toxicity, rehabilitation, cancer surveillance, cancer prevention and cancer survivorship (key aspects of supportive care). Clarity in understanding and application of terminology is therefore important; Table 1 includes definitions of associated terminologies, highlighting similarities and differences.

**Table 1:** Terminology relating to palliative care/supportive care.

Terminology	Definition
Palliative care	‘The active holistic care of individuals across all ages with serious health-related suffering due to severe illness and especially of those near the end of life’.^5^ See text for more detail.
Specialist palliative care	‘Provided by specialised services for patients with complex problems not adequately covered by other treatment options. Specialist palliative care services require a team approach, combining a multi-professional team with an interdisciplinary mode of work. Team members must be highly qualified and should have their main focus of work in palliative care’.^7^
Early palliative care	‘Begins at the time of, or shortly after, the diagnosis of advanced cancer. Often, early palliative care is combined with anticancer treatment such as chemotherapy or radiotherapy … involves empathetic communication with patients about their prognosis, advance care planning, and symptom assessment and control’.^11^
Timely palliative care	‘Early palliative care personalised around patients’ needs and delivered at the optimal time and setting’.^17^
Supportive care	‘The prevention and management of the adverse effects of cancer and its treatment’.^13^ See text for more detail.
Best supportive care	Historically used to describe the control arm in clinical trials in advanced cancer, but often referring to no specific care. Consensus guidelines for best supportive care standards are available, including multidisciplinary care, documentation, symptom assessment and management.^18^
Enhanced supportive care	Synonymous with early palliative care. An NHS England initiative ‘developed through recognition of what specialist palliative care can offer, but also from recognition of the barriers to achieving earlier involvement of palliative care expertise within the cancer treatment continuum’.^19^
Supportive oncology	Synonymous with supportive care. ‘Those aspects of medical care concerned with the physical, psychosocial and spiritual issues faced by persons with cancer, their families, their communities, and their healthcare providers. In this context, supportive oncology describes both those interventions used to support patients who experience adverse effects caused by antineoplastic therapies and those interventions now considered under the broad rubric of palliative care’.^20^

## Conclusion

Specialist palliative care has evolved somewhat over time, and will need to continue to evolve to maintain its relevance. Palliative care is not synonymous with supportive care, but is an important aspect of supportive care (and should be available to all cancer patients with relevant unmet needs). Equally, palliative care, both generalist and specialist, should, where relevant, be available to patients with other life-threatening non-malignant diseases.

## Funding

This article did not receive any specific grant from funding agencies in the public, commercial, or not-for-profit sectors.

## CRediT authorship contribution statement

**Amy Taylor:** Writing – review & editing, Writing – original draft. **Andrew Davies:** Writing – review & editing, Conceptualization.

## Declaration of competing interest

The authors declare the following financial interests/personal relationships which may be considered as potential competing interests:

Prof Davies is a member of the Editorial Board of *Clinical Medicine*. If there are other authors, they declare that they have no known competing financial interests or personal relationships that could have appeared to influence the work reported in this paper.
